# Development and validation of a computerized expert system for evaluation of automated visual fields from the Ischemic Optic Neuropathy Decompression Trial

**DOI:** 10.1186/1471-2415-6-34

**Published:** 2006-11-20

**Authors:** Steven E Feldon, Lori Levin, Roberta W Scherer, Anthony Arnold, Sophia M Chung, Lenworth N Johnson, Gregory Kosmorsky, Steven A Newman, Joanne Katz, Patricia Langenberg, P David Wilson, Shalom E Kelman, Kay Dickersin, members of the Ischemic Optic Neuropathy Decompression Trial Research Group

**Affiliations:** 1 Department of Ophthalmology, University of Rochester School of Medicine, Rochester, NY, USA; 2 Office of the Provost, University of Southern California School of Medicine, Los Angeles, Ca, USA; 3 Department of Epidemiology, Johns Hopkins University Bloomberg School of Public Health, Baltimore, Md, USA; 4 Department of Ophthalmology, Jules Stein Eye Institute, Los Angeles, Ca, USA; 5 Department of Ophthalmology, Saint Louis University Eye Institute, St. Louis, Mo, USA; 6 Department of Ophthalmology, University of Missouri's Mason Eye Institute, Columbia, Mo, USA; 7 Department of Neurophthalmology, Division of Ophthalmology, Cole Eye Institute, The Cleveland Clinic Foundation, Cleveland, Oh, USA; 8 Department of Ophthalmology, University of Virginia, Charlottesville, Va, USA; 9 Department of Ophthalmology, University of Maryland School of Medicine, Baltimore, Md, USA

## Abstract

**Background:**

The objective of this report is to describe the methods used to develop and validate a computerized system to analyze Humphrey visual fields obtained from patients with non-arteritic anterior ischemic optic neuropathy (NAION) and enrolled in the Ischemic Optic Neuropathy Decompression Trial (IONDT). The IONDT was a multicenter study that included randomized and non-randomized patients with newly diagnosed NAION in the study eye. At baseline, randomized eyes had visual acuity of 20/64 or worse and non-randomized eyes had visual acuity of better than 20/64 or were associated with patients refusing randomization. Visual fields were measured before treatment using the Humphrey Field Analyzer with the 24-2 program, foveal threshold, and size III stimulus.

**Methods:**

We used visual fields from 189 non-IONDT eyes with NAION to develop the computerized classification system. Six neuro-ophthalmologists ("expert panel") described definitions for visual field patterns defects using 19 visual fields representing a range of pattern defect types. The expert panel then used 120 visual fields, classified using these definitions, to refine the rules, generating revised definitions for 13 visual field pattern defects and 3 levels of severity. These definitions were incorporated into a rule-based computerized classification system run on Excel^® ^software. The computerized classification system was used to categorize visual field defects for an additional 95 NAION visual fields, and the expert panel was asked to independently classify the new fields and subsequently whether they agreed with the computer classification. To account for test variability over time, we derived an adjustment factor from the pooled short term fluctuation. We examined change in defects with and without adjustment in visual fields of study participants who demonstrated a visual acuity decrease within 30 days of NAION onset (progressive NAION).

**Results:**

Despite an agreed upon set of rules, there was not good agreement among the expert panel when their independent visual classifications were compared. A majority did concur with the computer classification for 91 of 95 visual fields. Remaining classification discrepancies could not be resolved without modifying existing definitions.

Without using the adjustment factor, visual fields of 63.6% (14/22) patients with progressive NAION and no central defect, and all (7/7) patients with a paracentral defect, worsened within 30 days of NAION onset. After applying the adjustment factor, the visual fields of the same patients with no initial central defect and 5/7 of the patients with a paracentral defect were seen to worsen.

**Conclusion:**

The IONDT developed a rule-based computerized system that consistently defines pattern and severity of visual fields of NAION patients for use in a research setting.

## Background

The Ischemic Optic Neuropathy Decompression Trial (IONDT) was a randomized clinical trial designed to test the safety and efficacy of optic nerve decompression surgery (ONDS) combined with careful follow-up for treatment of non-arteritic anterior ischemic optic neuropathy (NAION), as well as to document the natural history of NAION [1]. Using visual acuity as the primary outcome measure, the IONDT demonstrated that ONDS is not effective and may be harmful [2].

For NAION, characterized clinically as causing visual field loss, conclusions about treatment efficacy and natural history based on visual acuity outcomes alone may be inadequate. For this reason, change in the visual field, as measured by the Humphrey Visual Field Analyzer (HVF), was a planned secondary outcome in the IONDT. The Humphrey Visual Field Analyzer^® ^(Zeiss Humphrey, San Leandro, Ca, USA) provides a standardized testing environment, quantitative assessment of threshold sensitivity to spots of light at fixed points throughout the visual field, and data regarding reliability of patients' responses.

In the IONDT, we found no difference between visual fields from ONDS and careful follow-up groups at 6 months using the HVF global measure, "mean deviation" (MD). However, MD by itself may be an insufficient measure for assessment of visual fields in eyes with NAION. For example, the classical patterns of defect encountered in NAION may shift without changing average loss or there may be important changes in sensitivity within small areas of the visual field corresponding to nerve fiber bundle defects. These changes in area or size may not be detected when averaged into the MD measure, warranting a more detailed analysis of the quantitative visual field testing.

Development and validation of a system for classifying and assessing change in visual fields is complex due to the lack of "gold standards". Glaucoma trials have utilized a number of approaches for evaluating progression, but the algorithms seldom include classifications based upon the defect type. [3-6] Although the Optic Neuritis Treatment Trial (ONTT) investigators categorized visual field defects, they did not use strict definitions for classification and patterns of field loss were qualitatively rather than quantitatively determined [7].

Despite a variety of anticipated challenges, in the IONDT we set out to develop a rule-based computerized system for classifying and analyzing visual fields. Our intent was to create logic-based computer algorithms that reliably reproduced the clinical classifications of visual field defects encountered in NAION so as to evaluate the IONDT visual fields. The computer algorithm is intended for use in a clinical research setting where standardization of classification is required.

## Methods

### IONDT protocol

We have previously described the IONDT eligibility criteria, randomization procedure, and visit protocols in detail [1]. Briefly, patients aged 50 years or older were eligible for randomization into surgical or careful follow-up groups if they had symptoms and signs characteristic of NAION for 14 days or less in one eye. Patients with visual acuity of 20/64 or less in the study eye comprised the "regular entry" group, while patients otherwise eligible but with visual acuity better than 20/64 were enrolled only if visual acuity decreased to 20/64 or worse within 30 days of onset of symptoms ("late entry" group). Patients with acuity better than 20/64 and otherwise eligible and patients who refused randomization were followed as part of a non-randomized group. Institutional review boards at participating institutions approved the protocol and all participating patients provided signed informed consent.

We completed visual field testing of study and non-study eyes of all enrolled patients at baseline, for both randomized and non-randomized eyes. In the IONDT, automated perimetry was performed by trained certified visual field technicians using the HVF, 24-2 program with stimulus size III, full threshold strategy, and with foveal sensitivity measured concurrently. Visual fields for the study eye were measured before those for the non-study eye. For randomized patients, visual fields were obtained at the baseline examination; if randomization took place more than 24 hours after baseline, visual fields were re-measured. Clinical Centers measured visual fields prospectively. For randomized patients, this was at the 3, 6, and 12-month visit, at study closeout (minimum of 5 years of follow-up), and at approximately annual intervals between the 12-month and closeout visits. Visual fields for non-randomized patients were obtained at the baseline examination, at either 6 or 12-month visit or both, closeout, and at approximately one-year intervals between the 12-month and closeout visits. All patients were followed for at least 5 years. Visual field data were evaluated as a secondary outcome measure and not utilized for decision-making during the conduct of the trial.

### Methods used to develop the computerized visual field classification system

#### Development of classification system

In 1994, we formed a Visual Field Committee (VFC), which included an "expert panel" of six IONDT neuro-ophthalmologists (AA, SMC, SEF, LNJ, GK, SAN) with expertise in the interpretation of visual fields, five methodologists (KD, JK, PL, RWS, PDW), and a programmer (LL). The number of experts required on the panel was decided after a statistical computation determined that the chance of six experts agreeing on ten patterns by guessing would be 0.00001. A majority of the experts needed to agree to categorize a field defect as a specific pattern. The chance of this degree of concordance occurring by guessing alone was 0.01215. For any field in which the agreement among panelists was not significantly better than guessing, the field was considered 'non-classifiable'.

**Figure 1:**
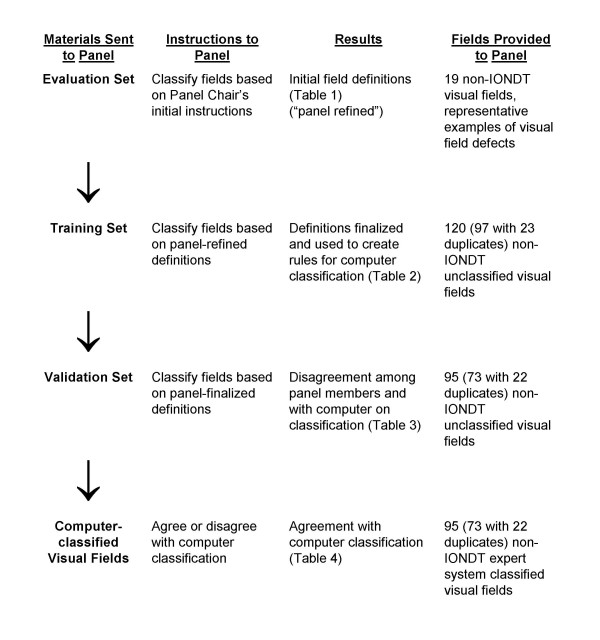
Sequence of steps utilized by the Visual Field Committee to develop rules for analysis of visual field defects by the computerized system.

The VFC established the protocol for developing visual field defect categories, training and evaluation of the expert panel, developing and testing of a computerized classification system, and defining progression using this system. The Committee based the sequence of steps that would be used to develop and validate the computerized expert system (see Figure 1) after Molino and associates [8]. The expert panel formulated the definitions of the various types of field defects, (e.g. normal, absolute defect, diffuse depression), all of which were based solely on data available within the 24-2 visual field.

We used 189 visual fields to develop the computer based classification system, none of which were associated with patients enrolled in the IONDT. Eighty-one visual fields were included from patients with NAION who were screened but not eligible for the IONDT. Reasons for ineligibility included refused enrollment, age < 50 years, onset of symptoms unknown or > 14 days, unable to read English, myocardial infarction within last 6 months, a visual condition precluding reliable visual acuity measurement, and current anticoagulant or corticosteroid use. One hundred eight visual fields from NAION patients not screened or enrolled in the IONDT, and seen at Doheny Eye Institute (n = 24), Emory University (n = 50), or the University of Missouri's Mason Eye Institute (n = 34) were also used to develop the computer-based system, following institutional review boards approval at each institution. All visual fields used to develop the computer-based classification system had been evaluated for reliability and had < 3 fixation losses, < 3 false positive responses, and < 3 false negative responses.

**Figure 2:**
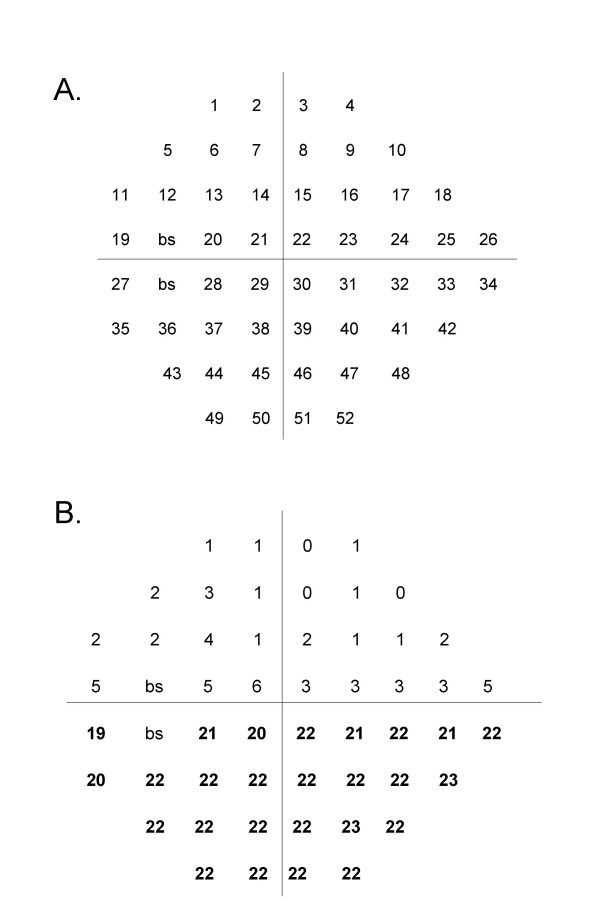
**A Schematic of a 52-point (program 24-2) Humphrey Visual Field for a left eye**. The point indicator number for each point measured is shown with its position in a visual field. "bs" indicates the two points that make up the blind spot. Points for the right eye are a mirror image, i.e., points are read right to left for each row. **B Example of a Humphrey Visual Field with an inferior altitudinal field defect**. This example of a visual field shows the difference between the values in decibels of each point in the linear array between a single visual field and those of age-matched controls. Differences in bold are those defining an inferior altitudinal defect.

The expert panel first formulated initial operational definitions of visual field defects corresponding to the 52 points in the 24-2 Humphrey scale (see Figure 2). Global guidelines included the following rules:

1. If a field is classified as normal or absolute (no perception of stimuli), no other classification may be made.

2. A depressed point is defined as equal to, or greater than, 4 dB loss.

3. Fields are classified even though they appear unreliable from the HVF indices (i.e., short term fluctuation).

4. Severity is based upon subjective judgment. Only the arcuate/altitudinal category may have more than one severity with a separate severity assignable to the arcuate and the altitudinal components.

Definitions were refined through an iterative process using an "evaluation set" of visual fields until consensus was reached, as follows:

The VFC director reviewed the 189 visual fields to select 19 with one or more representative defects (evaluation set), and then sent the evaluation set to each of the 6 expert panelists, along with instructions, a grading form, and proposed definitions for 13 types of defects and for levels of severity. Members of the expert panel independently reviewed the fields and definitions, and, after telephone and face-to-face meetings, agreed upon modified definitions of pattern defects.

The VFC Director used the modified pattern defect definitions to re-classify the 19 visual fields in the evaluation set. Each member of the expert panel independently reported the degree to which s/he agreed with the classification of each field, choosing from among the following choices: excellent, good, uncertain, poor, or bad. At the same time, the panelists were instructed to categorize the severity (density) of each defect as mild, moderate, severe, or absolute (Table 1). Because there was again lack of agreement among the expert panel on the classification, the group met face-to face to discuss and revise the existing definitions for a second time. Disagreements were resolved by allowing three categories of field defects: peripheral, paracentral, and central as well as a category of "other" which could be used only for visual fields that were impossible to fit into any other specific category.

**Table 1:** Agreement between six expert panel members' and director's classification for 19 non-IONDT visual fields in the Evaluation Set

	**Stated agreement with director**				
	**Excellent**	**Good**	**Uncertain**	**Poor**	**Bad**
	
**Director's classification**	**No. (%)**	**No. (%)**	**No. (%)**	**No. (%)**	**No. (%)**

Normal	5 (83)	1 (17)			
Absolute defect	6 (100)				
Mild diffuse depression	3 (50)	1 (17)		2 (33)	
Severe diffuse depression	1 (17)	4 (66)		1 (17)	
Mild superior altitudinal	3 (50)	3 (50)			
Moderate superior and inferior altitudinal		4 (67)	1 (17)		1 (17)
Severe superior altitudinal	6 (100)				
Mild inferior altitudinal	2 (33)	4 (67)			
Moderate inferior altitudinal	1 (17)	3 (50)		2 (33)	
Severe inferior altitudinal and moderate superior arcuate	5 (83)	1 (17)			
Moderate superior arcuate	4 (67)	1 (17)	1 (17)		
Severe superior and inferior arcuate	2 (33)	2 (33)	1 (17)		1 (17)
Mild inferior arcuate	6 (100)				
Moderate inferior arcuate	4 (67)	1 (17)	1 (17)		
Severe inferior arcuate	4 (67)	2 (33)			
Moderate inferior nasal step	5 (83)	1 (17)			
Mild paracentral scotoma	2 (33)		1 (17)	3 (50)	
Moderate central scotoma	4 (67)	2 (33)			
Severe central scotoma	3 (50)	3 (50)			

Using the revised definitions derived from the evaluation set, the VFC then sent a "training set" of 97 masked, representative, non-IONDT NAION fields to the expert panel for classification. To assess the ability of the panelists to apply the rules reliably, 11 duplicate fields from the training set and 12 fields from the evaluation set were included for a total of 120 fields.

At least five of six (83%) panelists independently agreed on the defect classification for 55 of 120 fields comprising the training set. Agreement on classification of the remaining 65 fields was achieved through a series of four interactive reconciliation meetings of the expert panel, held either by teleconference or in person. These discussions resulted in further refinement and finalization of the pattern definitions and consensus on classification of all fields in the training set.

The final classification system included "normal" and 13 different rule-based field or defect types, shown in Table 2. Severity was restricted to mild, moderate, and severe, and was defined subjectively.

**Table 2:** Panel-finalized definitions of visual field patterns and defects

**Type pattern or defect**	**Definition**
Normal	No quadrants depressed or only a few points in no specific pattern. One depressed point in a location surrounding the blind spot is normal unless it is part of another defined field defect.
Absolute defect	No response (sensitivity = zero) was recorded for all points in all quadrants or if only one point is less than or equal to 9 dB sensitivity and all other points are zero. If the retest is zero, then the point sensitivity is zero. Foveal sensitivity must be equal to zero.
Diffuse defect	Entire visual field equally depressed including fixation as defined as presence of both a superior and an inferior altitudinal defect that are equally depressed and a central scotoma
Superior altitudinal	Upper half of field equally depressed as defined as all points in the superior two quadrants approximately equally depressed, excluding those temporal to the blind spot (i.e. points 11 and 19 on the visual field map). Depression should extend down to horizontal meridian including approximate equal involvement of the superior paracentral points (points 21 and 22 on the visual field map).
Inferior altitudinal	Lower half of field equally depressed as defined as all points in the inferior two quadrants approximately equally depressed, excluding those temporal to the blind spot (i.e. points 27 and 35 on the visual field map). Depression should extend up to horizontal meridian including approximate equal involvement of the superior paracentral points (points 29 and 30 on the visual field map).
Superior arcuate	Peripheral defect (at least four peripheral points must be depressed within one quadrant) that appears in either or both superior quadrants with relative sparing of either one or both of the superior paracentral points, or either one of the superior paracentral points is less depressed in comparison to the superior periphery in either quadrant and it is not a nasal step. Superior periphery is defined as all points in the superior two quadrants except points 21 and 22.
Inferior arcuate	Peripheral defect (at least four peripheral points must be depressed within one quadrant) that appears in either or both inferior quadrants with relative sparing of either one or both of the inferior paracentral points, or either one of the inferior paracentral points is less depressed in comparison to the inferior periphery in either quadrant and it is not a nasal step. Inferior periphery is defined as all points in the inferior two quadrants except points 29 and 30.
Superior nasal step	An isolated superior nasal quadrant defect which preferentially involves the peripheral points (points 18,25, and 26) adjacent to the horizontal meridian. Cannot be part of a superior arcuate defect and there cannot be an arcuate defect in the superior temporal quadrant. Superior nasal points adjacent to the vertical meridian (points 3,8,15 and 22) are relatively spared.
Inferior nasal step	An isolated inferior nasal quadrant defect which preferentially involves the peripheral points (points 33,34 and 42) adjacent to the horizontal meridian. Cannot be part of an inferior arcuate defect and there cannot be an arcuate defect in the inferior temporal quadrant. Inferior nasal points adjacent to the vertical meridian (points 30, 39, 46, and 51) are relatively spared.
Central scotoma	Decreased sensitivity of the fovea by 5 dB relative to the least depressed point in the rest of the field or the foveal sensitivity is less than 10 dB.
Paracentral scotoma	Focal depression of the visual field not corresponding to any other pattern and located within the paracentral region (points 20,21,22,28,29,30) adjacent to the blind spot, but sparing fixation (i.e. no central scotoma). One isolated, depressed paracentral point next to the blind spot (point 20 or 28) is not a paracentral scotoma. If there is a central scotoma and, as defined, a paracentral scotoma, then the defect is categorized as a central scotoma.
Superior arcuate/altitudinal	Both superior paracentral points (points 21 and 22) are equally depressed, but the superior periphery is more depressed than the paracentral. Superior paracentral points must differ substantially from the inferior paracentral points (points 29 and 30) i.e. no central or paracentral scotoma involving these points.
Inferior arcuate/altitudinal	Both inferior paracentral points (points 29 and 30) are equally depressed, but the inferior periphery is more depressed than the paracentral Inferior paracentral points must differ substantially from the superior paracentral points (points 29 and 30) i.e. no central or paracentral scotoma involving these points
Other	Pattern defect that does not fit any of the above definitions e.g. shifted field. Use this category only if you are certain that you cannot categorize the defect using the other 13 categories

### The computer-based expert system

The VFC Director (SEF) and programmer (LL) translated the rules for the defect definitions and the general rules into a point-by-point set of algorithms applied using logical statements included with standard Excel software (computer-based expert system).

### Software structure

The computer-based expert system, constructed as a rule-based system on an Excel^® ^platform, run under Windows 98^®^, evaluated each field quadrant by quadrant. Quadrants were then analyzed in combination as needed to encompass definitions of all identified types of defects. The programmer translated each rule into a logical statement that could be found true or false, taking the form "if ... then". A truth table was utilized to define specific types of field defects, based upon definitions of the expert panel. Two forms of logical statements were used to identify pattern defects. The first statement was based upon *average *dB loss within a quadrant. If the average loss did not meet the criteria for depression (i.e., 4 dB) then the alternative statement, based on the *number *of disturbed points within a quadrant, was used to determine the presence of pattern defects. Thus, the number of disturbed points was used primarily to find mild defects that were missed by averaging.

For instance, if the average dB loss was greater in the periphery than in the central field by 5 dB, then an arcuate defect was defined as present in that quadrant (see definition in Table 2). If the central dB loss was greater by 5 dB than the periphery, then a central defect was present. If no pattern defect was found by averaging, then disturbed point algorithms were used to find mild or smaller pattern defects within a quadrant or defect-appropriate combination of quadrants. A fixed predetermined number of disturbed points had to be within the boundary of a pattern defect for that defect to be considered present. For example, a superior arcuate defect is defined as four depressed points within one or both upper quadrants (see definition in Table 2).

Some pattern defects were determined by the presence or absence of other defects. For example, if there were superior and inferior altitudinal defects and a central scotoma, then the pattern was defined as diffuse depression. If there was both a paracentral scotoma and a central scotoma, then the pattern was defined as a central scotoma alone.

Average dB loss within a pattern defect and number of disturbed points was used to classify a defect as mild, moderate, or severe. The severity classification of the expert panel was used to define the boundaries for each type of defect. Table 3 shows how severity for an altitudinal defect was determined using 23 altitudinal defects in the training set identified by the expert panel. Severity for other defects was similarly determined (number and type of defect used to determine severity scores: 9 paracentral scotomas; 26 arcuate defects; 20 diffuse depression defects; and 3 nasal step defects). Classification as an absolute defect (i.e. no response to the brightest stimulus at all points tested on the 24-2 HVF) required use of actual sensitivity rather than relative sensitivity loss.

**Table 3:** Classification of severity of 23 visual fields with altitudinal defects from training set

		**Field parameters**			
		
**Qualitative severity **(as classified by panel)	**n**	**Average dB loss within defect**	**95% CI**	**Average number points in defect**	**95% CI**
Mild	1	6.2		5	
Moderate	5	18.2	12.9 to 23.5	25	24.3 to 25.7
Severe	17	27.4	26.3 to 28.5	26	25.8 to 26.2

### Definition of change across time

#### Calculation of SF_C_

To measure change in visual field defects over time (i.e., from baseline to a follow-up visits) we planned to analyze visual fields at multiple time points and compare defect type and severity. We anticipated that change in an individual's visual field could entail change in defect type, defect severity, both defect type and severity, identification of a new defect at follow-up not observed at baseline, or disappearance of a defect observed at baseline.

Because spurious changes in visual fields caused by patient learning effects or by short term fluctuation were possible, we decided to use the Humphrey global index short term fluctuation (SF), a measure of intra-test variability, as a standard by which to determine the normal variation within an individual's visual fields over a fixed time period. SF is determined by measuring the difference between two threshold measurements at 10 different points across the entire field during the same test. The average of these differences constitute the SF. Clinically, a small SF (1 to 2 dB) indicates a reliable field [9]. To estimate the normal variation of an individual's visual fields measured at baseline and follow-up, we used a pooled estimate, SF_C_, for both visits, calculated as follows:

SFC=1.96[(SFbaseline2+SFfollowup2)]
 MathType@MTEF@5@5@+=feaafiart1ev1aaatCvAUfKttLearuWrP9MDH5MBPbIqV92AaeXatLxBI9gBaebbnrfifHhDYfgasaacH8akY=wiFfYdH8Gipec8Eeeu0xXdbba9frFj0=OqFfea0dXdd9vqai=hGuQ8kuc9pgc9s8qqaq=dirpe0xb9q8qiLsFr0=vr0=vr0dc8meaabaqaciaacaGaaeqabaqabeGadaaakeaacqWGtbWucqWGgbGrdaWgaaWcbaGaem4qameabeaakiabg2da9iabigdaXiabc6caUiabiMda5iabiAda2maadmaabaWaaOaaaeaacqGGOaakcqWGtbWucqWGgbGrdaqhaaWcbaGaemOyaiMaemyyaeMaem4CamNaemyzauMaemiBaWMaemyAaKMaemOBa4MaemyzaugabaGaeGOmaidaaOGaey4kaSIaem4uamLaemOray0aa0baaSqaaiabdAgaMjabd+gaVjabdYgaSjabdYgaSjabd+gaVjabdEha3jabdwha1jabdchaWbqaaiabikdaYaaakiabcMcaPaWcbeaaaOGaay5waiaaw2faaaaa@5694@

where SF_C_, is half of the 95% confidence interval on the pooled estimate of SF across both visits, SF_baseline _is the SF measured for the visual field at baseline and SF_follow-up _is the SF measured at follow-up (i.e., from the visual field obtained at the 6 month visit for determining change from baseline to the 6 month visit or from the visual field obtained at the 12 month visit for change from baseline to the 12 month visit).

When there was an apparent change in defect type from baseline to follow-up we removed the effect of normal variation by using the value of SF_C _to "adjust" the follow-up visit Humphrey visual field at key points used by the computerized expert system to differentiate between defects types. The adjustment was made in the direction that would decrease the probability of detecting a change in defect type from baseline to follow-up. The adjusted data was then reclassified. For example, if an individual had visual fields classified as having a superior arcuate defect at baseline and a superior altitudinal defect at the follow-up visit, that patient's SF_C _for these visits was subtracted from the points that distinguish an arcuate from an altitudinal defect in the computerized expert system, i.e., paracentral points 21 and 22, in the follow-up field. This adjusted follow-up visual field was then re-classified. If the adjusted visual field was still classified as having a superior altitudinal defect, then the superior portion of the follow-up field would be classified as having changed from baseline, from a superior arcuate to a superior altitudinal defect. On the other hand, if the adjusted follow-up visual field was classified as having a superior arcuate defect, then the follow-up visual field was classified as "not changed" with a superior arcuate defect for both visits. This general approach was used to distinguish an arcuate from an altitudinal defect, and a central from a paracentral defect.

Change in severity was also determined after applying SF_C _but was only evaluated in fields whose defects were classified as not changed.

## Results

### Validation of the classification scheme

A set of 95 non-IONDT NAION visual fields was sent to the expert panel as a "validation set"; of these, 22 were masked duplicates chosen systematically from the original training set (every fifth field listed in ID numeric order). The level of agreement on classification of these fields among the expert panel and the corresponding agreement of the computer with the panel members' classifications is shown in Table 4. Reliability of individual panel members in re-classifying defects in the 22 duplicate visual fields from the evaluation set averaged 57% (range; 32% to 77%), despite a common set of definitions derived and finalized by consensus.

**Table 4:** Agreement among members of the expert panel and agreement of computer with expert panel in independent classification of visual fields in the validation set

	**95 visual fields in validation set**			
	
	**Fields for which expert panel agrees on classification**		**Fields for which computer classification agrees with majority of panel members**	
	
**Expert panel members agreeing on classification**	**No**.	**(%)**	**No**.	**(%)**
6 of 6 panelists agree	7	(7)	7	(7)
5 of 6 panelists agree	14	(15)	10	(11)
4 of 6 panelists agree	23	(24)	16	(17)
3 of 6 panelists agree	22	(23)	16	(17)
**Total fields for which ≥ 3 panelists agree**	**66**	**(69)**	**50**	**(53)**
**Total visual fields**	**95**	**(100)**	**95**	**(100)**

**Figure 3:**
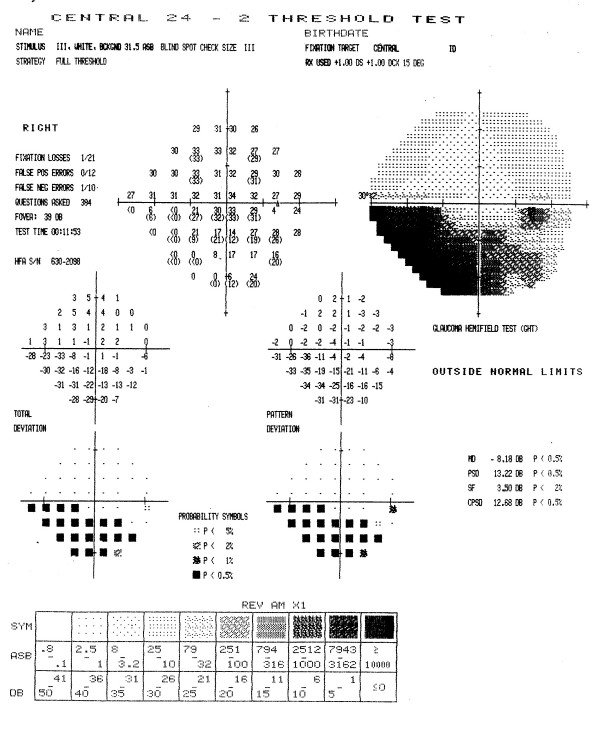
**Example of visual field associated with excellent agreement among members of the expert panel and computerized classification**. All members of the expert panel independently classified the defect in this visual field as a moderate inferior arcuate scotoma, and the computer algorithm applied the same classification. In addition, all members of the expert panel concurred with the computerized classification when asked if the computer classification was a valid clinical classification.

**Figure 4:**
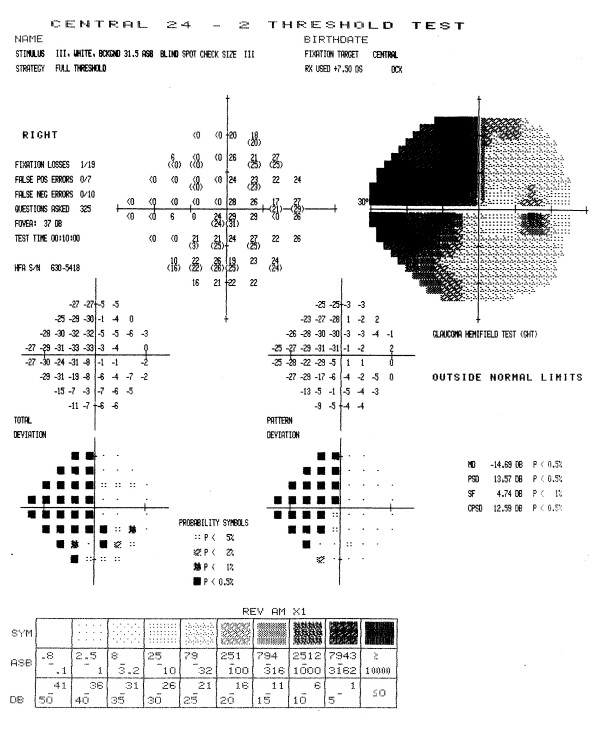
**Example of good agreement among members of the expert panel, and moderate agreement with computer classification**. Five members of the expert panel classified the defects in this visual field as a moderate superior arcuate and a moderate inferior arcuate, while the other member classified the defects as a moderate nasal step and inferior arcuate. None agreed with the computer classification, moderate superior arcuate and severe inferior nasal step. Five members of the panel concurred with the computerized defect pattern classification, although only three agreed with the severity classification. Dissenting members thought that defects were either both severe or both moderate. The remaining member thought that the defects were best described as moderate superior and inferior arcuate scotomas.

**Figure 5:**
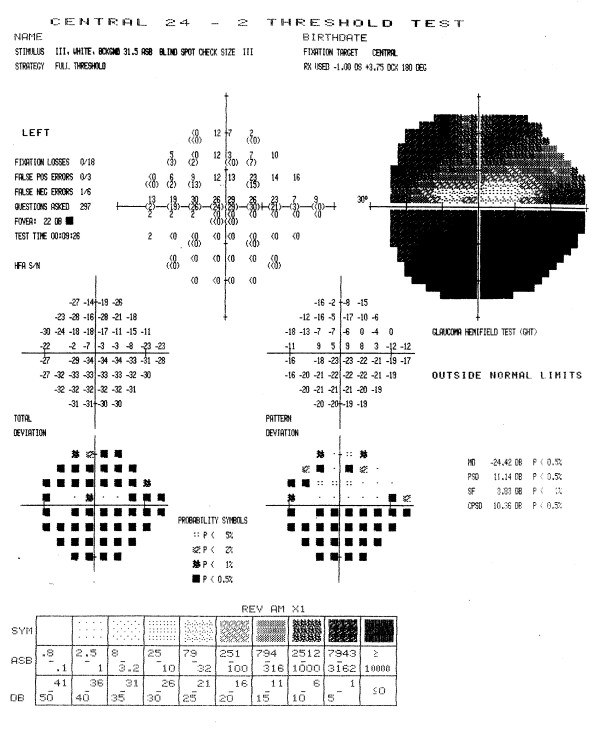
**Example of poor agreement among members of the expert panel, but agreement with computer classification**. Three members of the expert panel initially and independently classified the defects in this visual field as a moderate superior arcuate and severe inferior altitudinal scotoma. One member classified the superior defect as an arcuate-altitudinal, while two other members thought this visual field also had a moderate central scotoma in agreement with the computerized classification. When asked if the computer classification was a valid clinical classification, all members of the expert panel concurred with the computerized classification (moderate superior arcuate, severe inferior altitudinal and moderate central scotoma).

**Figure 6:**
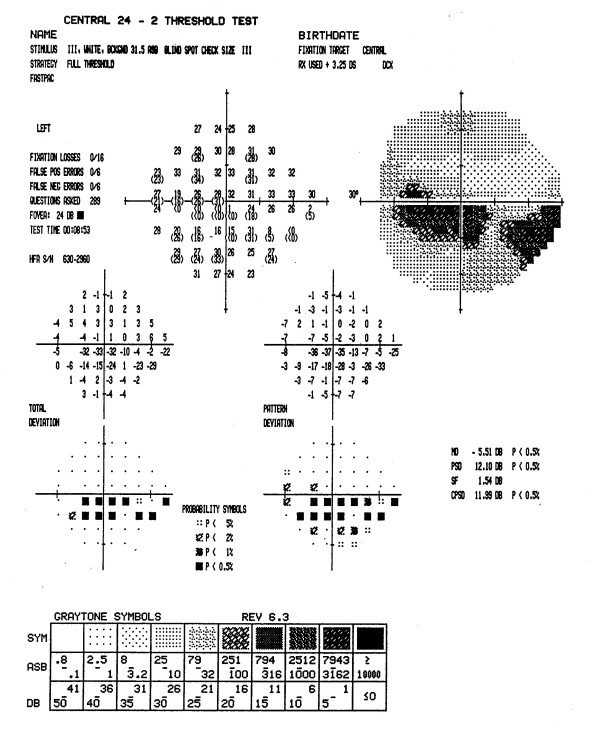
**Example of poor agreement among members of the expert panel, and good agreement with computer classification**. No member of the expert panel initially and independently classified the defects in this visual field in exactly the same way as any other member. Classifications included a mild inferior altitudinal with or without a moderate central scotoma and with or without a mild superior arcuate, or as a paracentral scotoma with or without a mild to moderate inferior arcuate scotoma. When asked if the computer classification was a valid clinical classification, all members of the expert panel concurred with the computerized classification (mild to moderate inferior arcuate and moderate central scotoma).

**Figure 7:**
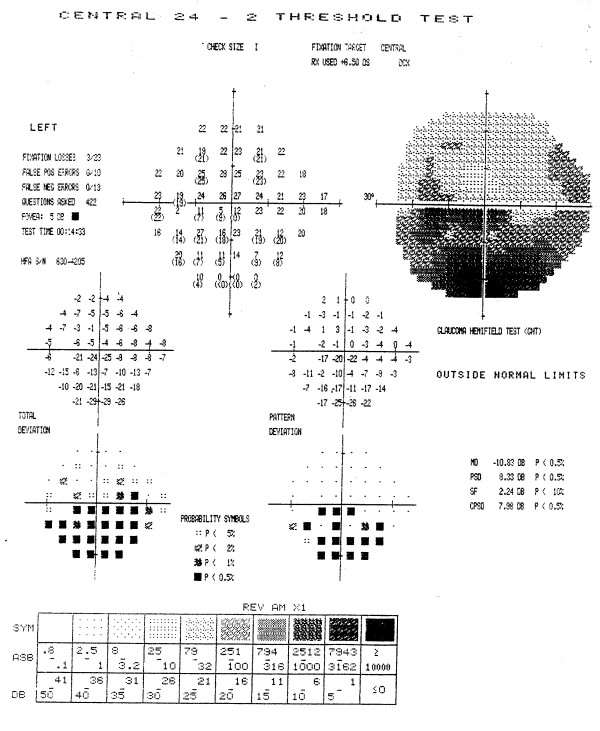
**Example of poor agreement among members of the expert panel, and poor agreement with computer classification**. Only two members of the expert panel initially and independently classified the defects in this visual field in exactly the same way, i.e., as a mild superior altitudinal, moderate inferior altitudinal, and severe central scotoma. Other classifications included a superior arcuate, inferior arcuate or paracentral scotoma. Only two members of the panel concurred with the computer classification (mild superior arcuate, moderate inferior altitudinal, and severe central scotoma). Two members believed that the inferior defect was an arcuate rather than altitudinal defect, one member believed that the field represented overall diffuse depression with a superior arcuate.

Figures 3 through 7 show representative visual fields used in the validation process and illustrate the type of disagreement that was found. Figure 3 shows an example of visual fields for which the expert panel members independently arrived at pattern and severity classifications that were exactly the same as the computerized classification. Figure 4 shows a visual field in which the members agreed among themselves but not with the computer classification, and Figures 5, 6 and 7 show fields for which there was little agreement among the expert panel during independent classification.

We then used an alternative validation approach, whereby the panelists were asked to agree or disagree with the computer's classification. We changed the question posed to panel members from one of application of the rules to classify the defects in this visual field to "does the consistent application of consensus-derived rules applied by the computer program result in a classification of this visual field that is clinically acceptable?" There were only 4 of 95 instances in which the majority (≥ 50%) of the panelists did not believe that the computer classification was clinically acceptable (Table 5). Specific differences were

**Table 5:** Agreement with computer-based classification of 95 visual fields in the validation set among six members of the expert panel

	**Visual field**	
	
**In agreement with computer**	**No**.	**(%)**
Six of six panelists agree	59	(62)
Five of six panelists agree	24	(25)
Four of six panelists agree	8	(8)
Three of six panelists agree	2	(2)
Two of six panelists agree	2	(2)
**Total visual fields**	**95**	**(100)**

(1) Identification of an additional mild superior altitudinal defect by the computer, but not the panel members for one field;

(2) Classification by the computer of one field as having a severe diffuse defect and as a combination of three separate defects (superior arcuate, inferior arcuate, and central scotoma) by the expert panel;

(3) Classification by the computer as an altitudinal or arcuate defect and by the expert panel as an arcuate or altitudinal, respectively in 2 visual fields.

Figure 7 is an example of the third type of disagreement listed. Although two members of the panel concurred with the computer classification (superior arcuate, inferior altitudinal, and central scotoma), two members classified the inferior defect as an arcuate and one member classified the superior defect as an altitudinal defect. One member believed that only a superior arcuate was present. Investigation revealed that if the computer algorithm were modified to allow concordance with the panel members, other classification errors would result; therefore, these discrepancies were allowed to stand. Thus, there was majority agreement of the expert panel and the computer classification in 91 of 95 (96%) fields.

### Validation of change

To test our approach to define change, we examined the "change" in the study eye visual field from baseline to the randomization visit for IONDT late entry patients. It is reasonable to expect that a majority of late entry patients experienced a change in the central visual field in addition to the measured change in visual acuity. Table 6 shows the unadjusted number and type of central defects observed in visual fields of 47 IONDT late entry patients at the baseline and randomization visits. Using data without any adjustment for normal variation, we found that 14 of 22 (63.6%) of the patients who had neither a paracentral nor central defect at baseline developed a central defect by the randomization visit. In addition, all 7 patients who started with a paracentral defect developed a central defect by the randomization visit. Of 18 patients starting with a central defect, only one changed to a paracentral defect at randomization.

**Table 6:** Comparison of defects in central location of study eye at baseline visit with defects at randomization visit, in late entry patients, without correction for short term fluctuation (SF_C_)

	**Defect at randomization visit (RV)**							
	
	**Paracentral**		**Central**		**Neither paracentral nor central**		**Total**	
**Defect at baseline visit**	**No. (%)**	**% at RV**	**No. (%)**	**% at RV**	**No. (%)**	**% at RV**	**No. (%)**	**% at RV**

**Paracentral**	0	0	7 (100.0)	18.4	0	0	7 (100.0)	14.9
**Central**	0	0	17 (94.4)	44.7	1 (5.6)	11.1	18 (100.0)	38.3
**Neither paracentral nor central**	0	0	14 (63.6)	36.8	8 (36.4)	88.9	22 (100.0)	46.8
**Total**	0	0	38 (80.8)	100.0	9 (19.2)	100.0	47 (100.0)	100.0

**Figure 8:**
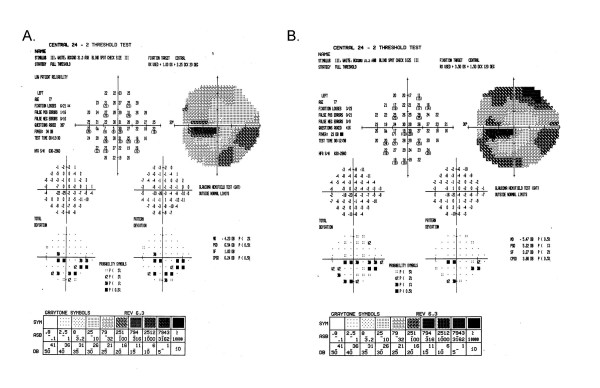
**Change in visual field from baseline to randomization for a late entry study participant**. The superior field showed no defect at baseline (8A). Nine days later at the randomization visit (8B), it was classified as an arcuate and remained as an arcuate after adjustment using SF_C_. The inferior field was classified as an arcuate at both baseline and at randomization and so no adjustment using SF_C _was applied. The central field was classified as a paracentral at baseline, and a central at randomization, but remained a paracentral defect after adjustment using SF_C_.

**Figure 9:**
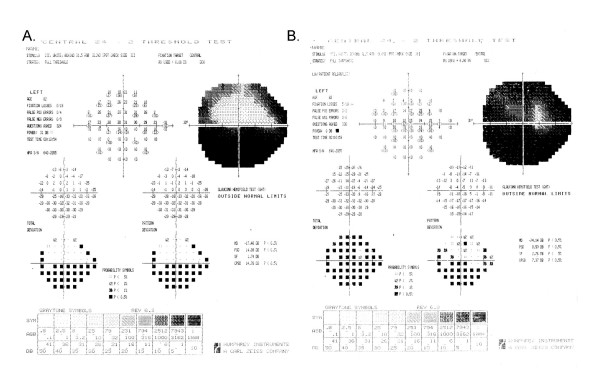
**Change in visual field from baseline to randomization for a late entry study participant**. The superior field showed an arcuate defect at baseline (9A). Twenty-one days later, at the randomization visit (9B), it was classified as an altitudinal but after adjustment using SF_C_, it remained classified as an arcuate defect. The inferior field was classified as an arcuate at baseline and worsened to an altitudinal defect at randomization both before and after adjustment using SF_C_. The central field was classified as a paracentral at baseline, and as a central scotoma at randomization both before and after adjustment using SF_C_.

When we applied the adjustment, SF_C_, for each patient's normal variation to the visual fields of the late entry eyes, the classification of 2/47 randomization fields was different. Five rather than the initial 7 patients who had a paracentral defect at baseline had a central defect at randomization (see Table 7). All other defect changes remained the same. A Stuart-Maxwell chi-square test of homogeneity showed that the shift in distribution of defects from baseline to randomization as shown in Table 7 is statistically significant (p = 0.0003). There was no observed change in severity (average dB loss) for the central defect of the 17 study participants who had a central defect at both baseline and randomization (mean 11.5 dB versus 6.7 dB at baseline and randomization, respectively; p = 0.09) after SF_C _adjustment. Figures 8 and 9 show examples of visual fields obtained at baseline and randomization visits in two late entry IONDT study participants; these examples show the type of change detected by the computerized system.

**Table 7:** Comparison of defects in central location of study eye at baseline visit with defects at randomization visit, in late entry patients, with correction for short term fluctuation (SF_C_)

	Defect at randomization visit (RV)*							
	
	**Paracentral**		**Central**		**Neither paracentral nor central**		**Total**	
**Defect at baseline visit**	**No. (%)**	**% at RV**	**No. (%)**	**% at RV**	**No. (%)**	**% at RV**	**No. (%)**	**% at RV**

**Paracentral**	2 (28.6)	0	5 (71.4)	13.9	0	0	7 (100.0)	14.9
**Central**	0	0	17 (94.4)^†^	47.2	1 (5.6)	11.1	18 (100.0)	38.3
**Neither paracentral nor central**	0	0	14 (63.6)	38.9	8 (36.4)	88.9	22 (100.0)	46.8
**Total**	2 (4.3)	100.0	36 (76.6)	100.0	9 (19.2)	100.0	47 (100.0)	100.0

* p = 0.0003; Stuart Maxwell test for marginal homogeneity

† p = 0.09 for change in severity, 11.5 dB at baseline versus 6.7 dB at randomization; paired t test

## Discussion

Automated perimetry facilitates the collection of quantitative data on the pattern and severity of visual field defects. To date, however, full use has not been made of quantitative data for detection, characterization, or progression of visual field defects in ischemic optic neuropathy. In the IONDT, we developed and validated a reliable rule-based system. This system proved capable of consistently defining pattern and severity of visual field defects detected in eyes with NAION enrolled in the IONDT. It also allowed for evaluation of change in visual field defect and severity over time. Development of this system entailed devising definitions for visual field defects encountered in NAION patients using an iterative process and expert panel and defining progression. All decision rules were based upon the opinion of the expert panel; these rules then provided the basis by which all field classifications were made. Further testing of the system showed that this rule-based computer program is a valid method for analysis of the patterns and severities of visual field data for individuals with NAION.

Development and validation of a system for classifying visual fields is complex, given that there is no existing "gold standard" for defect classification and that experts are unable to reach agreement on defect classification, at least in this study. This type of problem is well known in medicine. For instance, studies validating the use of computer-assisted diagnosis tools [8,10,11] suggest that the differences between computer diagnosis and human expert diagnosis differed by about the same extent as human experts disagree among themselves. The diagnostic variability in this study was similar to performance of humans and computers in validations of other expert systems for interpretations lacking a "gold standard", for which agreement ranged from 50% to 70% [8,10,11]. Given that computerized diagnosis may be no better than that of an expert panel, the principal reason for utilizing a computerized system in the context of a clinical trial is to reduce inconsistency by eliminating intra- and inter-observer variability. For example, we found that members of the expert panel often did not classify a visual field the same way they previously classified it. Thus, use of a computerized system reduces variability, although not necessarily the original bias of the expert panel in classification of visual field defects. Once incorporated into a computer system, the criteria for categorizing the pattern and severity of visual field defects are, according to Hirsbrunner and colleagues [12], "explicit, obvious, and standardized." Such attributes are essential within the context of randomized clinical trials.

Development and validation of a classification system for visual fields requires several steps [8,10,11]. First, an expert panel must achieve consensus on a set of rules for classifying defects. Second, the experts apply the rules successfully, i.e., the rate of agreement was not meaningfully different from agreement reported for similar classification systems in other medical contexts. Third, the consistent application of the rules by a computerized system produces classifications that do not disagree with the panel more than the expert panel disagrees with itself. Finally, the computerized system produces reasonable defect classifications, defined as classifications with which the expert panel rarely disagrees.

We recognized that more than one interpretation was possible for a given distribution of disturbed points on a visual field and that it was not going to be possible for all the experts to agree on a gold standard to evaluate the computerized system. Thus, we elected to accept the computerized determination given that the panel considered it to be consistent with clinical interpretation.

A quantified or computerized analysis of visual fields that approximates a human interpretation of an automated visual field faces particular challenges in three areas – detection, progression, and characterization of the defect. Difficulties in detection of defect relate primarily to distinguishing appropriately between short-term and long-term fluctuation. This problem is further compounded in various disease states, such as glaucoma, in which the pathological process itself produces fluctuation in sensitivity [13]. The Ocular Hypertension Treatment Study used multiple confirmation fields to diagnose the presence or absence of a defect and provides an example of a method to deal with clinical detection of visual field defects [14]. More advanced models of visual field perturbations, such as those by De la Rosa and colleagues [15], utilize an approach for rapid assessment of glaucomatous field defects based upon multiple correlations. Although the IONDT computerized system cannot distinguish between short and long-term fluctuation when detecting a defect pattern within a single field, it does use a standard set of rules for classification and detection and thus provides for consistent identification and classification of defects.

Progression of field defects is a common end-point for glaucoma studies. The issue, once again, is determining change, but from an abnormal as opposed to a normal baseline. Katz [16] reviewed scoring methods employed by two multicenter clinical trials, the Advanced Glaucoma Intervention Study (AGIS) and the CIGTS. These studies utilized a cumulative score (0–20), based upon depression of adjacent points occurring within specified regions of the visual field. Depression was defined by total deviation plot on the HVF printout in the AGIS and by probability values in the CIGTS. McNaught and co-workers [17] developed a linear model of point wise sensitivity values against time to identify progression in normal tension glaucoma. By any of these methods, detection and progression could be determined operationally, based on the sensitivity and reliability required in a particular study. The IONDT used change, defined as decibel loss or increased number of points within defects identified at baseline, to detect progression using the computerized classification system, after adjusting for measured within-individual variations in performance.

In contrast to detection and progression of visual field defects, characterization is a more complex task. It requires pattern recognition open to multiple interpretations and preferences (e.g., "lumping" versus "splitting"). Typically, glaucoma visual field interpretation does not address visual field characterization. In one of the few clinical trials to utilize pattern recognition as an outcome for visual field testing, the Optic Neuritis Treatment Trial (ONTT) established 15 monocular types of field defects (14 local and diffuse) of three different severities occurring in optic neuritis [18]. The Director and Associate Director of the ONTT Visual Field Reading Center reviewed visual fields separately, then together, to "reach a consensus on the final classification for each visual field." Initial agreement was noted for 76.3% of the HVF, 81.5% on the location and 74% on the shape. Complete agreement in every category was achieved in only 47.4% of 309 affected eyes. In a masked retest, the agreement on shapes was present for 76.2% of 42 cases [7,18]. The same investigators have recently developed a similar classification methodology for visual fields obtained in the Ocular Hypertension Treatment Study (OHTS). Complete agreement in classification among three readers was achieved in 64%–66% of defects and majority agreement was achieved in an additional 31%–33% [19].

Other methods have been used to characterize visual fields. For example, neural networks have been touted as providing a means for allowing computers to "learn" how to categorize visual fields correctly, even in the absence of specified rules. In the supervised class of artificial neural networks, the systems require a training set of "correctly" categorized visual fields to allow learning to occur [20-23]. Thus, there is a tautology in that, in the absence of rules, how is such a training set derived? Henson and associates suggest that unsupervised neural networks can be used to resolve this dilemma, as they are self-classifying [24]. However, the patterns correspond to the number of nodes used in the neural network and do not necessarily correspond to clinically identified field defects.

In designing the computerized system for evaluation of IONDT visual fields, we encountered several methodological issues that could have influenced definitions of defect classification and/or change. First, fields were obtained using full threshold strategy rather than SITA, which resulted in prolonged testing times. SITA strategies were unavailable at the outset of patient recruitment and had not yet been completely validated by the study end. Second, because the IONDT did not formally train study patients on performing Humphrey visual field exams before collecting study visual field data, some observed changes may be due to learning effects over time. The importance of training was not generally recognized in 1992, when the IONDT began. However, the testing method used in the IONDT is probably generalizable, given that most patients in a clinical setting do not undergo visual field training sessions. Despite these methodological issues, the observed changes in pattern classification and severity of IONDT visual fields were remarkably consistent over time [25], suggesting that it is unlikely that the computer system algorithms had substantial classification errors.

Another concern relating to study validity was the failure of the 6 experts to agree completely on a sizable proportion of defect classifications for the test fields during the initial validation. The number of experts we included in our testing differed substantially from those utilized in virtually all other prospective trials involving visual fields. This was a deliberate decision to ensure rigor and avoid chance agreement. The observed lack of concordance in classifying defects by the 6 experts is most likely due to the number of experts (6 experts rather than the usual 2 experts plus a tie-breaker) and independence of reviewers (experts from geographically dispersed clinical centers). Indeed, we believe members of our expert panel were more likely to manifest true independence in decision-making than experts at a single reading center.

## Conclusion

In summary, we developed a computerized method for analyzing automated perimetric data in the IONDT. This system is a validated rule-based system capable of consistently defining pattern and severity of visual field defect encountered in NAION patient. Its primary use is in the research setting; these methods are not meant to replace clinical evaluation. Once incorporated into a computer system, the criteria for categorizing the pattern and severity of visual field defects are explicit, obvious, and standardized. Such attributes are essential within the context of randomized clinical trials.

## Abbreviations

AGIS: Advanced Glaucoma Intervention Study

CIGTS: Collaborative Initial Glaucoma Treatment Study

HVF: Humphrey Visual Field Analyzer

IONDT: Ischemic Optic Neuropathy Decompression Trial

MD: mean deviation, a Humphrey Visual Field Analyzer global index

NAION: non-arteritic anterior ischemic optic neuropathy

OHTS: Ocular Hypertension Treatment Study

ONDS: optic nerve decompression surgery

ONTT: Optic Neuritis Treatment Trial

SF: short term fluctuation, a Humphrey Visual Field Analyzer global index

SF_C_: the pooled estimate of short term fluctuation across two visits

SITA: Swedish Interactive Thresholding Algorithm

VFC: Visual Field Committee for the Ischemic Optic Neuropathy Decompression Trial

## Competing interests

The author(s) declare that they have no competing interests.

## Authors' contributions

SEF conceived the computer expert system, contributed to the design of the development and validation studies, chaired the expert panel, interpreted the study findings, and contributed to writing and editing the manuscript. LL coordinated activities of the expert panel, wrote the algorithm for the computer system. RWS contributed to the design of the development and validation studies, coordinated the validation studies, contributed to interpreting the study findings, and contributed to writing and editing the manuscript. AA collaboratively developed the algorithms for identification of the visual field patterns and participated in the validation studies as a member of the expert panel. SMC collaboratively developed the algorithms for identification of the visual field patterns and participated in the validation studies as a member of the expert panel. LNJ collaboratively developed the algorithms for identification of the visual field patterns and participated in the validation studies as a member of the expert panel. GK collaboratively developed the algorithms for identification of the visual field patterns and participated in the validation studies as a member of the expert panel. SAN collaboratively developed the algorithms for identification of the visual field patterns and participated in the validation studies as a member of the expert panel. JK contributed to the design of the development and validation studies, interpretation of study findings. PL contributed to the design of the development and validation studies, provided statistical advice, and contributed to writing and editing the manuscript. PDW contributed to the design of the development and validation studies, provided statistical advice, and contributed to writing and editing the manuscript. SEK served as scientific Chair of the Ischemic Optic Neuropathy Decompression Trial, contributed to the design of the study. KD contributed to the design of the development and validation studies, interpreted the study findings, and contributed to writing and editing the manuscript. All authors read and approved the final manuscript.

## Pre-publication history

The pre-publication history for this paper can be accessed here:


